# Reliability and construct validation of the Blended Learning Usability Evaluation–Questionnaire with interprofessional clinicians in Canada: a methodological study

**DOI:** 10.3352/jeehp.2025.22.5

**Published:** 2025-01-16

**Authors:** Anish Kumar Arora, Jeff Myers, Tavis Apramian, Kulamakan Kulasegaram, Daryl Bainbridge, Hsien Seow

**Affiliations:** 1 Office of Education Scholarship, Department of Family and Community Medicine, University of Toronto, Toronto, ON, Canada; 2 Family Medicine Education Research Group, McGill University, Montreal, QC, Canada; 3 The Wilson Centre, Temerty Faculty of Medicine, University of Toronto, Toronto, ON, Canada; 4 Juravinski Cancer Centre, Hamilton, ON, Canada; 5 Department of Oncology, McMaster University, Hamilton, ON, Canada; The Catholic University of Korea, Korea

**Keywords:** Educational measurement, Medical education, Program evaluation, Reproducibility of results, Validation study, Canada

## Abstract

**Purpose:**

To generate Cronbach’s alpha and further mixed methods construct validity evidence for the Blended Learning Usability Evaluation–Questionnaire (BLUE-Q).

**Methods:**

Forty interprofessional clinicians completed the BLUE-Q after finishing a 3-month long blended learning professional development program in Ontario, Canada. Reliability was assessed with Cronbach’s α for each of the 3 sections of the BLUE-Q and for all quantitative items together. Construct validity was evaluated through the Grand-Guillaume-Perrenoud et al. framework, which consists of 3 elements: congruence, convergence, and credibility. To compare quantitative and qualitative results, descriptive statistics, including means and standard deviations for each Likert scale item of the BLUE-Q were calculated.

**Results:**

Cronbach’s α was 0.95 for the pedagogical usability section, 0.85 for the synchronous modality section, 0.93 for the asynchronous modality section, and 0.96 for all quantitative items together. Mean ratings (with standard deviations) were 4.77 (0.506) for pedagogy, 4.64 (0.654) for synchronous learning, and 4.75 (0.536) for asynchronous learning. Of the 239 qualitative comments received, 178 were identified as substantive, of which 88% were considered congruent and 79% were considered convergent with the high means. Among all congruent responses, 69% were considered confirming statements and 31% were considered clarifying statements, suggesting appropriate credibility. Analysis of the clarifying statements assisted in identifying 5 categories of suggestions for program improvement.

**Conclusion:**

The BLUE-Q demonstrates high reliability and appropriate construct validity in the context of a blended learning program with interprofessional clinicians, making it a valuable tool for comprehensive program evaluation, quality improvement, and evaluative research in health professions education.

## Graphical abstract

[Fig f1-jeehp-22-05]

## Introduction

### Background/rationale

Blended learning is increasingly recognized as a pivotal educational paradigm in the field of health professions education [1-4]. Blended learning programs are educational interventions that integrate synchronous learning modalities (e.g., real-time, in-person or videoconferencing sessions) with asynchronous learning modalities (e.g., pre-recorded online modules and learning management systems) [5,6]. In so doing, blended learning programs offer more flexible and personalized learning experiences compared to traditional face-to-face or fully online learning modalities [1-6]. Learners engaged in blended learning describe better control over the content, sequence, pace, and timing of their learning, often leading to more meaningful educational experiences [1-6]. Educators adopting a blended learning approach can teach knowledge-building content (i.e., memorization-focused material) through asynchronous modules, and skill-building content (i.e., practical experienced-based learning) in synchronous sessions [3-6]. This flexibility can enhance learner engagement, satisfaction, and educational outcomes [1-4].

Despite their benefits, evaluations of blended learning programs in health professions education remain haphazard, hindering quality improvement, scaling, and systematic comparisons [5]. Challenges around evaluation can be attributed to the fact that evaluative terminology is often undefined and poorly conceptualized across health professions education (e.g., some studies may consider increase in learner satisfaction as sufficient evidence for effectiveness of a program, whereas others may only consider increase in post-intervention test scores as evidence for effectiveness) [5]. Additionally, though questionnaires are the most utilized approach to evaluating blended learning programs in health professions education, most are often not designed or validated for the purpose of evaluating such programs (e.g., many use their institution’s generic end-of-course questionnaire as a baseline measure for program evaluation) [5]. Recently, evaluation scholarship has turned toward the construct of “usability” to support comprehensive and meaningful evaluations of blended learning programs [5,6].

Usability, as perceived by learners, is a multidimensional construct which encompasses the following domains: effectiveness, efficiency, satisfaction, accessibility, organization, and overall experience from engaging with a product, technology, and/or service [6]. Thus, usability goes beyond simply measuring “ease of use” to, instead, comprehensively evaluating the quality of systems, products, and services [5,6]. Although usability has been highly utilized for evaluating e-learning programs, its utilization with blended learning programs has been poor [5,6]. This is potentially due to the added complexity of blended learning programs (i.e., content spread across different learning modalities), as opposed to more straightforward evaluations of online learning settings (e.g., accessibility and organization of a learning management system).

To enable rigorous usability-focused evaluations of blended learning programs in health professions education, the Blended Learning Usability Evaluation–Questionnaire (BLUE-Q) was developed [6,7]. To date, content validity (i.e., if items are understandable, meaningful, comprehensive, and if sufficient item-domain correlation exists) for the BLUE-Q has been established through a Bayesian questionnaire validation approach with medical and health science faculty members [7]. However, other evidence for the BLUE-Q’s construct validity (i.e., the degree to which a tool measures the theoretical construct it intends to assess) [8,9] including reliability evidence (i.e., the degree to which a tool is free from random error) [9,10], as established through real-world application of the BLUE-Q with learners, remains unexplored.

Importantly, in recent years, the use of mixed methods has gained traction as an effective approach to establishing construct validity [8]. Specifically, by integrating quantitative and qualitative data, a more comprehensive understanding can be generated around how the underlying theoretical construct of the tool being validated is conceptualized and rated by users. This breadth of data overcomes limitations from traditional validation methods which are primarily quantitative, and thus, often fall short in capturing the nuances and contextual factors that are critical to understanding a construct’s full meaning and relevance across diverse settings and populations [8]. Including mixed methods construct validity with reliability evidence improves the depth of evaluation of educational tools by working towards both statistical rigor and real-world applicability.

### Objectives

The purpose of this study is to collect construct validity and reliability evidence for the BLUE-Q to verify its potential to accurately evaluate usability of blended learning programs based on the perceptions of interprofessional clinical learners.

## Methods

### Ethics statement

Ethics approval to conduct this study was obtained from the University of Toronto Research Ethics Board (Protocol #: 00046242). All participants signed consent forms prior to engaging in the study.

### Study design

This study adopts a convergent design where quantitative and qualitative data were collected and analyzed concurrently [11].

### Setting

The setting for this study was a continuing professional development program addressing serious illness communication skills. The “All providers: Better Communication Skills” (ABCs) is a blended learning program consisting of synchronous skill-building workshops conducted virtually, and a companion set of asynchronous e-learning modules delivered over 3 months. Recruitment, piloting, and program evaluation for the ABCs was conducted in Ontario, Canada between January to May 2024.

### Participants

Participants were palliative care professionals (e.g., physicians, nurses, social workers, etc.) who had signaled a desire to develop communication skills through a provincial palliative care professional development registry.

### Variables

The BLUE-Q is a mixed-methods questionnaire with 3 sections: (1) pedagogical usability (e.g., evaluation of the program’s content, learning objectives, and experience of learners with their instructors); (2) usability of the synchronous learning components (e.g., evaluation of face-to-face sessions); and (3) usability of the asynchronous learning components (e.g., evaluation of recorded modules and learning management systems) [6,7].

### Data sources/measurement

Participants completed the BLUE-Q at the ABCs program completion. The BLUE-Q is comprised of 23 five-point Likert scale items and 6 open-ended items (i.e., each section has 1 item addressing overall thoughts and 1 item that requests suggestions for improvement) [7]. To ensure that BLUE-Q items are understood by learners in this context, some items were slightly revised for phrasing specificity (e.g., “face-to-face component” was replaced with “virtual workshops”). Research data are available at Dataset 1.

### Bias

Learner perceptions of the quality of educational interventions can sometimes be influenced by the quantitative assessments they receive [12]. To mitigate this potential bias, participants were not given their final assessment scores, and were only given feedback for improvement after they completed the BLUE-Q. Another potential bias can be attributed to participant selection. Given that participants were highly motivated to participate in this program, as identified through their registration, a potential to rate this program more favorably exists. However, use of both quantitative and qualitative items addressing participants’ experience with various aspects of the program enables a comprehensive and rigorous approach to evaluating perspectives.

### Study size

There was no sample size estimation. All 50 participants were initially enrolled in the program and 40 completed all program components, including the BLUE-Q.

### Statistical methods

All quantitative data were analyzed on IBM SPSS Statistics ver. 29.0 (IBM Corp.). Descriptive statistics, including means and standard deviations were calculated for each Likert scale item and by each questionnaire section. Cronbach’s α was calculated for each of the 3 BLUE-Q sections and for all quantitative items together [9,10]. In general, a Cronbach’s α value above 0.7 is considered acceptable, and a value above 0.8 is preferable [10].

The framework by Grand-Guillaume-Perrenoud et al. [8] was used to guide a mixed methods approach to collecting construct validity evidence. In this framework, congruence, convergence, and credibility are considered critical elements of construct validity [9]. Congruence refers to the relationship between the content of the item and the corresponding open-ended response (i.e., are qualitative responses on-topic, off-topic, or unclear?) [9]. Convergence refers to the level of agreement between quantitative ratings and qualitative responses (i.e., do quantitative and qualitative data converge, diverge, or is this unclear or neutral?) [9]. Credibility refers to a classification of what the qualitative response is trying to convey (i.e., is the response confirming, disconfirming, or clarifying quantitative ratings?) [9]. Specifically, the first author independently reviewed the congruence, convergence, and credibility between raters’ qualitative and quantitative responses, and then discussed and validated these with the second author. A table was generated which includes: the mean ratings and standard deviation for each BLUE-Q quantitative item; 5 illustrative qualitative excerpts exemplifying congruence, convergence, and credibility by questionnaire section; and Cronbach’s α for each section of the questionnaire.

## Results

### Participants

The 40 learners were comprised of: 5 physicians or nurse practitioners; 17 nurses; 9 social workers; 2 health educators, 1 spiritual care provider; and 6 healthcare administrators, coordinators, or directors. Three learners identified as male and 37 identified as female.

### Main results

Mean rating (with standard deviations) for the pedagogy, synchronous learning, and asynchronous learning sections were 4.77 (0.506), 4.64 (0.654), and 4.75 (0.536) respectively. Cronbach’s α for the pedagogical section was 0.95, for the synchronous learning section was 0.85, for the asynchronous learning section was 0.93, and for all quantitative items was 0.96.

Almost every participant responded to all 6 open-ended items (i.e., 239 out of 240 possible responses were received). Among the 239 qualitative responses, 61 were considered non-substantive (i.e., these responses were some forms of “no comment”), leaving 178 substantive comments for analysis. Of the 178 substantive comments, 22 were considered off-topic because in 2 cases participants were asked to provide their overall perceptions of the program, but instead only provided a suggestion for improvement, and in 20 cases they were asked to provide suggestions for improvement, but instead only provided positive comments about the program. The rest of the 156 comments were considered on-topic as there was no mismatch between the response provided and what was asked. As 156 (88%) of the substantive responses were on-topic, high congruence can be inferred.

The 156 on-topic substantive responses were classified into the following categories: only positive comments (n=107), positive comments given alongside a description of a challenge experienced during the program (n=5), only a description of a challenge experienced during the program (n=3), positive comments given alongside some type of suggestion for program improvement (n=8), and only a suggestion for improvement (n=33). On-topic substantive responses that only included a positive comment (107/156, 69%) were considered confirming statements and all other responses (49/156, 31%) were considered clarifying statements, which together suggests appropriate credibility.

To further explore credibility among the 41 on-topic responses that included a suggestion for program improvement, the following categories of recommendations were identified: improving access to resources, for example, by enabling learners to download module content (n=8); addressing issues with logistics such as fixing an issue regarding logging in on the learning management system (n=3); improving modular content, for example, by adding more content (n=9); revising the structure and setting of workshops through, for example, increasing the length (n=18); and improving the facilitation and evaluative process through, for example, providing more detailed feedback at the end of the course (n=3).

Finally, given that across all 178 substantive responses, 140 included a positive comment (79%), adequate convergence is identified between qualitative comments and the high numeric ratings across each section of the BLUE-Q. See Table 1 and 2 for a summary of the main results.

## Discussion

### Key results

The BLUE-Q demonstrated strong reliability evidence as a tool for evaluating blended learning programs by clinical learners through high internal consistency across all sections of the tool. Additionally, through adopting the validation framework by Grand-Guillaume-Perrenoud et al. [8], evidence of high congruence, good convergence, and appropriate credibility were identified, suggesting acceptable construct validity. Lastly, after thorough analysis of qualitative responses, the BLUE-Q was able to elicit key recommendations of quality improvement for future iterations of the ABCs program.

### Interpretation

As expressed through this study, the BLUE-Q is an efficient tool for a comprehensive blended learning program evaluation. In the case of the ABCs program, quantitative ratings indicate high perceived usability with regards to the pedagogy, synchronous learning modality, and asynchronous learning modality. However, it can be noted that the synchronous learning section received the lowest ratings, especially around the efficiency and satisfaction domains. Through the BLUE-Q’s qualitative data, it can be understood that though participants felt the synchronous learning experience was exceptionally important in helping them improve their skills, they had wished for more time to practice and more feedback to assist in their learning. Thus, as a mixed methods tool, the BLUE-Q appears capable of guiding educators toward reliable insights for continual improvement and refinement of educational interventions.

### Comparison with previous studies

The content validity of the BLUE-Q was previously established through a Bayesian approach with faculty members [7]. However, a key limitation in that study was that reliability and other validity evidence through application of the BLUE-Q with learners could not be generated [7]. This article fills that gap. Additionally, construct validity is often one of the most poorly understood and under-evaluated aspects of validity evidence [13]. However, in this study, the mixed methods framework by Grand-Guillaume-Perrenoud et al. [8] provides a clear approach to conceptualizing and analyzing qualitative and quantitative data to generate construct validity evidence. Furthermore, existing tools that evaluate blended learning programs in medical education often focus on measuring student perceptions around satisfaction [14] or focus particularly on the e-learning aspects of a blended program [15]. The BLUE-Q, however, garners insights from learners on several usability domains (e.g., effectiveness, efficiency, satisfaction, etc.) across the content, synchronous, and asynchronous aspects of blended learning programs.

### Limitations

This study had a small sample size, but the overall consistency between quantitative ratings and qualitative data suggests high utility for the BLUE-Q in the clinical education context.

### Generalizability

The participants of this study were diverse in terms of their professional roles, but limited in terms of their gender (92.5% women). However, the mixed methods construct validation approach proved to be useful and relevant for assessing the relationship between quantitative ratings and open-ended qualitative responses obtained for the BLUE-Q. Future studies with larger and more diverse samples may assist in further addressing generalizability.

### Suggestions

This study confirms the utility of the BLUE-Q for program evaluations across health professions education, suggesting it can be used widely in this field. Given that blended learning program evaluations in the context of health professions education are often challenged by the lack of conceptual and methodological rigor and consistency (e.g., non-standardized and non-reliability-tested department-specific program evaluation measures) [4], the BLUE-Q can serve as an instrument to further quality improvement and evaluative research in this context. This is particularly valuable in the rapidly changing landscape of health professions education, where pedagogical approaches continue to evolve to better serve clinical learners.

For time-sensitive evaluations using the BLUE-Q, focus on quantitative ratings may be sufficient for gauging any major challenges across content, synchronous, and asynchronous modalities. When pursuing a more thorough understanding of learner perspectives, specifically around what aspects of the program are well or poorly received, and recommendations for change, integration of qualitative data with quantitative data is critical. Application of the BLUE-Q early into the program can assist in gauging any major challenges that learners are encountering with the intervention, which can then be addressed in a timely manner. Application at the end of a program supports outcome evaluations around perceived satisfaction, effectiveness, efficiency, accessibility, organization, and overall learner perceptions.

### Conclusion

In conclusion, the BLUE-Q is a highly reliable and valid tool for blended learning program evaluations with interprofessional healthcare learners. Its mixed methods approach and comprehensive assessment provides valuable insights for program improvements, guides rigorous evaluative research, and contributes to the broader goal of enhancing rigor in the field of health professions education.

## Figures and Tables

**Figure f1-jeehp-22-05:**
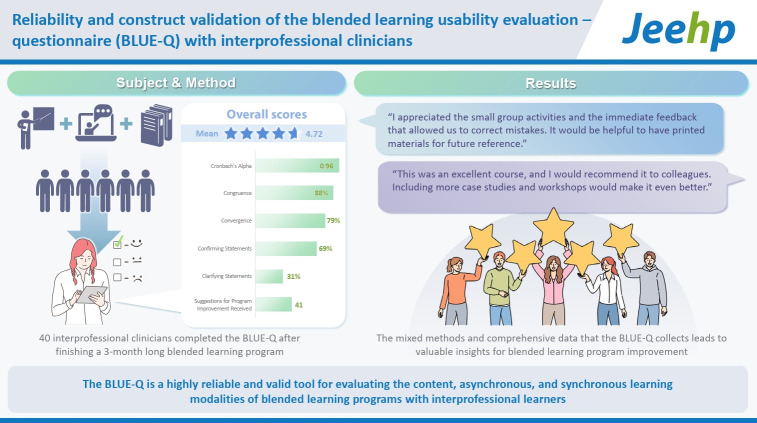


**Table 1.** Summary of results 1

BLUE-Q domains	BLUE-Q items	Mean±SD by item
Part 1: Pedagogical usability		
Effectiveness	1. The content taught in this course helped me gain new knowledge (i.e., facts or information) and/or strengthen previously acquired knowledge.	4.8±0.46
	2. The content taught in this course helped me gain new skills (i.e., ability to perform specific tasks) and/or strengthen previously acquired skills.	4.85±0.43
	3. The content taught in this course corresponds to the learning objectives discussed in the introduction of the course (e.g., learn a set of principles and approaches that guide skillful communication about serious illness).	4.78±0.48
	4. The assessments (e.g., activities in the modules to check knowledge, simulations in the workshop to practice skills) in this course were helpful for my learning.	4.78±0.53
	5. Overall, I learned a lot from this course.	4.78±0.53
Efficiency	6. The amount of work required for this course (i.e,. online modules and workshops combined) was manageable.	4.6±0.55
	7. The instructor was available to answer my questions during the workshop.	4.73±0.64
Satisfaction	8. I enjoyed learning the content in this course.	4.78±0.48
	9. I was motivated to learn the content in this course.	4.78±0.48
Accessibility & organization	10. The content of this course was delivered in a way that made sense to me.	4.78±0.48
Part 2: Usability of the synchronous learning modality		
Effectiveness	11. Being face-to-face with the teacher in the online workshops helped me learn the course content.	4.68±0.57
Efficiency	12. The amount of time we spent in the face-to-face online workshops was appropriate.	4.43±0.87
Satisfaction	13. I enjoyed the face-to-face online workshops.	4.58±0.68
	14. I felt motivated to attend the face-to-face online workshops.	4.68±0.57
Accessibility & organization	15. The face-to-face online workshops of this course were easy-to-access.	4.68±0.76
	16. The material taught in the face-to-face online workshops was well organized.	4.8±0.46
Part 3: Usability of the asynchronous learning modality		
Effectiveness	17. The online modules helped me learn.	4.7±0.79
Efficiency	18. The amount of time I spent completing online modules was appropriate.	4.65±0.53
Satisfaction	19. I enjoyed the online modules.	4.73±0.55
	20. I felt motivated to complete the online modules.	4.73±0.51
Accessibility & organization	21. The online modules for this course were easy-to-access on my technological devices.	4.78±0.48
	22. The online modules were easy to navigate.	4.8±0.46
	23. The online modules were well organized.	4.85±0.43

BLUE-Q, Blended Learning Usability Evaluation–Questionnaire; SD, standard deviation.

**Table 2.** Summary of results 2

BLUE-Q domains	Excerpts exemplifying congruence, convergence, and credibility by questionnaire part	Cronbach’s α by questionnaire part
Part 1: Pedagogical usability	“I have gained so many insights on communication principles, especially in the context of serious illness conversations, which I believe is crucial in my role as a nurse.” (Participant 4)	0.95
	“I feel this course gave me some great tools to be mindful about when I am communicating with patients and family members during difficult prognoses and decision making. I feel that it was a good course to really look at how we talk and actively listen to patients and family members and can be used in all conversations when providing patient care.” (Participant 12)	
	“The content was provided very structured and easy to follow.” (Participant 13)	
	“The course was great! Good pace, left enough time between learning modules and online sessions for people that are working full time.” (Participant 22)	
	“I really enjoyed this training and learned a lot and reinforced my existing skills. Thank you again for providing this wonderful training.” (Participant 23)	
Part 2: Usability of the synchronous learning modality	“Very well organized and easy to follow.” (Participant 1)	0.85
	“Great to have the virtual meetings—easy to attend and participate in the course.” (Participant 14)	
	“I felt the instructor was great during the workshops and I did learn a lot from them. They were able to convey worked experiences to us making the content more interesting.” (Participant 18)	
	“I really enjoyed the face-to-face and small group size of this training. It was engaging and very helpful.” (Participant 23)	
	“The online workshops were very well done. The pace was excellent...” (Participant 29)	
Part 3: Usability of the asynchronous learning modality	“Modules were well organized and thorough.” (Participant 6)	0.93
	“I enjoyed the asynchronous online learning. I felt it was separated into manageable sections and the content was useful.” (Participant 17)	
	“I’m not very technical but I found the platform easy to navigate” (Participant 22)	
	“I found the online modules to be organized, clear, concise and easy to learn from.” (Participant 34)	
	“Being able to do the modules before the face to face was great as it helped with learning the process and then being able to put it into practice.” (Participant 36)	

BLUE-Q, Blended Learning Usability Evaluation–Questionnaire.

## Footnotes

**Data availability**
Data files are available from Harvard Dataverse: https://doi.org/10.7910/DVN/ZZPUWB Dataset 1. An excel document which includes all anonymized BLUE-Q data from the 40 learners.

## Supplementary materials

Supplement 1. Audio recording of the abstract.
